# Efficient Multi-Target Localization Using Dynamic UAV Clusters

**DOI:** 10.3390/s25092857

**Published:** 2025-04-30

**Authors:** Wei Gong, Shuhan Lou, Liyuan Deng, Peng Yi, Yiguang Hong

**Affiliations:** 1 Department of Control Science and Engineering, Tongji University, Shanghai 201804, China; weigong@tongji.edu.cn (W.G.); 2232948@tongji.edu.cn (S.L.); dengliyuan99@tongji.edu.cn (L.D.); yipeng@tongji.edu.cn (P.Y.); 2 Shanghai Research Institute for Intelligent Autonomous Systems, Tongji University, Shanghai 201210, China

**Keywords:** multi-target localization, clustered UAV systems, combinatorial optimization, dynamic clustering, quantum-inspired optimization

## Abstract

This paper proposes a dynamic unmanned aerial vehicle (UAV) clustering model for multi-target localization in complex 3D environments, where mobility-aware cluster formation is integrated to enhance collaborative localization accuracy. We derive the Cramér–Rao lower bound (CRLB) for localization performance analysis under measurement and motion-induced uncertainties. To solve the NP-hard clustering problem, we develop the MDQPSO-ASA algorithm, which combines multi-swarm discrete quantum-inspired particle swarm optimization with adaptive simulated annealing, incorporating a repair mechanism to satisfy spatial and cardinality constraints. Simulation results demonstrate the algorithm’s superiority in localization accuracy, computational efficiency, and adaptability to varying UAV/target scales compared to baseline methods. The developed algorithm provides an effective solution for resource-constrained collaborative localization tasks in practical scenarios.

## 1. Introduction

With the rapid development of unmanned aerial vehicle (UAV) technology, utilizing dynamic UAV networks for collaborative localization of multiple targets has become an important research topic. This technology demonstrates significant application potential in areas such as disaster rescue [1], wildlife monitoring [2], and infrastructure inspection [3]. However, due to the limited resources of UAV networks, insufficient individual sensing capabilities, and the wide distribution of targets, constructing an effective clustering model to achieve wide-area collaborative localization presents a critical challenge. In this context, it is necessary to design a clustering scheme that comprehensively considers the dynamic movement of UAVs, sensing resource allocation within the cluster, and the requirements of localization tasks.

The problem of UAV clustering for collaborative localization involves using multiple mobile UAVs to locate several potential non-cooperative targets (NCTs) in 3D environments. In this process, the UAVs, which are constantly moving, are divided into several clusters, each acting as a collaborative unit to localize the targets. This dynamic clustering problem aims to achieve optimal overall localization performance while accounting for the continuous motion of UAVs. Although extensive research has been conducted on target localization and wireless sensor network clustering separately, very few studies have systematically integrated UAV clustering strategies with multi-target localization tasks, particularly in scenarios where UAVs are mobile. Therefore, this paper aims to fill this research gap by exploring how to significantly improve the accuracy and efficiency of multi-target localization through adaptive clustering strategies and efficient collaboration mechanisms.

Traditional clustering methods often focus on optimizing specific metrics, such as minimizing energy consumption, reducing communication latency, maximizing coverage, or balancing nodes within clusters. However, in the context of multi-UAV collaborative localization, optimizing these single objectives often fails to meet the demands of real-world tasks. The focus of this paper is on improving the accuracy of multi-target localization through collaboration among multiple UAVs. Specifically, the goal of clustering is not only to achieve rational resource allocation but also to ensure that UAVs within each cluster can collaborate efficiently, fully leveraging their respective sensing and communication capabilities to achieve high-precision localization of potential NCTs in complex 3D environments. Additionally, algorithm design must consider real-time performance and robustness in dynamic environments to address uncertainties such as target movement and communication quality.

In summary, this paper addresses the challenge of multi-target localization using dynamic UAV clusters by proposing a novel clustering model that accounts for UAV mobility and resource constraints. The model integrates mobility-aware cluster formation and communication-efficient quality metrics to enhance localization accuracy in complex 3D environments. The proposed MDQPSO-ASA algorithm combines multi-swarm optimization, discrete quantum-inspired particle swarm optimization, and adaptive simulated annealing to solve the NP-hard clustering problem efficiently. Simulation results validate the superior performance of the proposed approach in terms of solution quality and computational efficiency.

The main contributions of this paper are summarized as follows:

- We propose a novel dynamic clustering model for multi-target localization using UAV clusters, which accounts for UAV mobility and resource constraints. This model utilizes multiple UAVs in different clusters to collaboratively localize NCTs, enhancing localization accuracy and operational maneuverability in complex 3D environments.
- We derive the Cramér–Rao lower bound (CRLB) for the proposed clustered localization framework, incorporating measurement uncertainties and motion-induced errors. This theoretical analysis provides critical insights into the achievable localization accuracy under specific cluster configurations.
- We propose the MDQPSO-ASA algorithm, which integrates multi-swarm optimization, discrete quantum-inspired particle swarm optimization, and adaptive simulated annealing. Additionally, a repair mechanism is introduced to ensure the feasibility of the solutions by enforcing spatial and cardinality constraints. This algorithm effectively addresses the NP-hard clustering problem, ensuring high-quality solutions and computational efficiency.
- We conduct extensive simulation experiments to validate the performance of the proposed algorithm. Through comparisons with baseline algorithms, we show that MDQPSO-ASA consistently achieves better solution quality and faster convergence. Besides, the results demonstrate the superior performance of the MDQPSO-ASA algorithm in terms of solution quality, computational efficiency, and adaptability to different scales of UAVs and NCTs.

The rest of this paper is organized as follows: Section 2 reviews related work in localization and clustering techniques. Section 3 presents the system model, including the dynamic UAV network architecture, mobility-aware quality metrics, and CRLB derivation. Section 4 details the proposed MDQPSO-ASA algorithm. Section 5 evaluates the performance of the proposed algorithm through simulation experiments. Finally, Section 6 concludes the paper.

## 2. Related Work

In this section, we review recent advancements in localization and clustering techniques and identify gaps that motivate our work.

### 2.1. Localization

In terms of target localization, major research and engineering practices typically employ range-based localization algorithms [4]. These algorithms include time of arrival (TOA), time difference of arrival (TDOA), direction of arrival (DOA), and received signal strength (RSS). The performance of these algorithms is typically evaluated using the classical Cramér–Rao lower bound (CRLB) [5], which provides a theoretical lower bound for the mean-square error (MSE) of unbiased estimators. However, as application scenarios become increasingly complex, traditional single-model approaches often struggle to achieve high localization accuracy.

Consequently, recent studies have shifted toward improving localization performance not only by integrating multiple measurement models or optimizing system design but also by leveraging emerging physical-layer technologies. For instance, the exploration of the terahertz (THz) band in 6G networks [6,7] has demonstrated remarkable potential in enhancing localization accuracy. With its ultra-wide bandwidth and high spatial resolution, the THz band surpasses the capabilities of mmWave-based systems [8], paving the way for transformative improvements in localization capabilities. Besides, Panwar et al. [9] proposed a hybrid TOA-RSS localization algorithm that enhances target localization accuracy under non line of sight (NLOS) conditions by solving a weighted least squares problem. Similarly, Wang et al. [10] addressed the issue of NLOS errors in indoor 3D TOA-based localization by proposing an error mitigation method. This approach estimates and corrects ranging errors and calculates the target position using linear least squares, significantly improving localization accuracy.

In parallel, sensor deployment strategy has emerged as a critical factor influencing localization performance. Xu et al. [11] investigated the optimal placement of sensors in 3D space based on the TOA model, applying the A-optimality criterion (i.e., minimizing the trace of the inverse Fisher information matrix (FIM)) to determine the best sensor positions under Gaussian noise conditions, thereby minimizing localization errors. Additionally, Zhao et al. [12] addressed the sensor placement optimization problem for TDOA-based localization in cluttered indoor environments using ultra-wideband signals, employing the block coordinate-wise minimization (BCM) algorithm [13] to solve the problem.

Nevertheless, these studies are largely confined to “single-target, multi-sensor” localization models and do not meet the requirements for cooperative localization in large-scale scenarios. There remain notable gaps in addressing the challenges of complex environments and large-scale cluster-based cooperative localization.

### 2.2. Clustering

Clustering has been a widely studied area, with significant advancements made in improving the performance of wireless sensor networks (WSNs) and multi-agent systems. For instance, Han et al. [14] introduced a meta-heuristic-based clustering protocol to extend network lifetime and enhance throughput, while Verma et al. [15] focused on securing data transmission in intelligent transportation systems through an innovative clustering method. Similarly, Guo et al. [16] developed the ICRA intelligent clustering routing algorithm to improve routing stability and reduce packet loss during data transmission. Additionally, El Khediri et al. [17] presented an energy-efficient *K*-means clustering algorithm for WSNs, prioritizing energy consumption as a key metric to prolong network operation under spatial constraints. Beyond WSNs, clustering has also been applied to multi-UAV systems. Wu et al. [18] addressed task allocation for UAV networks in large-scale interception scenarios, leveraging clustering to coordinate UAV groups effectively. Chen et al. [19] proposed an adaptive clustering approach for UAV path planning, optimizing time efficiency and task completion rates in heterogeneous multi-UAV missions.

Despite significant advancements in clustering, most existing studies primarily focus on traditional objectives, such as network formation, energy efficiency, and task allocation. Some research has explored target localization using UAV clusters, with notable contributions in this area. Arafat et al. [20] proposed particle swarm optimization (PSO)-based swarm intelligence localization (SIL) and swarm intelligence clustering (SIC) algorithms to address the localization and clustering challenges in UAV networks. Their work demonstrated improvements in localization accuracy while reducing energy consumption. However, their approach relies on the assumption that UAVs operate in static configurations, thereby overlooking the challenges arising from dynamic mobility To address this gap, recent studies have begun incorporating dynamic models into UAV localization frameworks. For instance, Garraffa et al. [21] proposed a closed-loop control system for mobile targets by integrating robotic methodologies, demonstrating stable tracking in dynamic scenarios. Nevertheless, these methods primarily focus on single-target tracking and lack scalable collaboration mechanisms for UAV clusters operating in complex 3D environments In contrast, our work aims to develop novel clustering strategies and collaboration mechanisms tailored to the dynamic nature of UAV networks, addressing the unique challenges posed by UAV mobility. This enables more accurate and robust localization in complex 3D environments, where both spatial dynamics and multi-agent coordination must be simultaneously considered.

## 3. System Model

In this section, we establish a cooperative localization model integrating UAV mobility patterns and estimation-theoretic analysis. The proposed system model consists of four fundamental components: (1) a dynamic UAV network-based localization architecture with cluster-based coordination mechanisms, (2) mobility-aware and communication-efficient quality metrics for cluster formation, (3) CRLB analysis incorporating both measurement uncertainties and motion-induced errors, and (4) an optimization formulation for cluster assignment of UAVs.

### 3.1. System Architecture and Basic Definitions

In this work, we investigate the cooperative localization problem of *M* NCTs with a UAV network consisting of *N* UAVs distributed in 3D space, where N≥M ensures system observability. The UAVs are represented by the set $U={⋃}_{n=1}^{N}\left\{u_{n}\right\}$, while the NCTs are denoted by set $G={⋃}_{m=1}^{M}\left\{g_{m}\right\}$. The position of UAV $u_{n}$ and NCT $g_{m}$ is represented by 3D vectors $p_{n}\in R^{3}$ and $q_{m}\in R^{3}$, respectively. Besides, $v_{n}\in R^{3}$ denotes the velocity of $u_{n}$.

**Figure 1:**
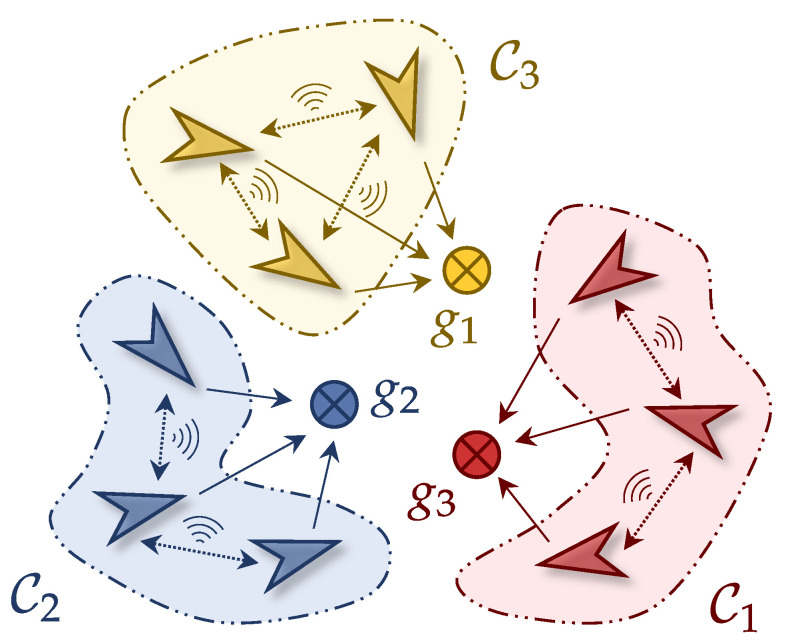
An example scenario of dynamic UAV clusters for multi-target localization, where 9 UAVs are divided into 3 clusters $C_{1}$, $C_{2}$, $C_{3}$, while 3 NCTs $g_{1}$, $g_{2}$, $g_{3}$ are assigned to the clusters, respectively.

To enhance localization accuracy, we adopt a cluster-based architecture where the UAVs are partitioned into *K* distinct clusters ${\left\{C_{k}\right\}}_{k=1}^{K}$ collaboratively estimating the position of a designated NCT $g_{m}\in G$ with each cluster $C_{k}\subseteq U$. The cardinality of each cluster, denoted by $|C_{k}|$, represents the number of UAVs in $C_{k}$. Figure 1 illustrates an example scenario of dynamic UAV clusters for multi-target localization. UAVs in the same cluster are expected to localize the same NCT while communicating with each other.

Let $x_{n,k}\in \left\{0,1\right\}$ to denote whether UAV $u_{n}$ is clustered into $C_{k}$, where

(1) $$x_{n,k}=\left\{\begin{array}{cc} 1 & \mathrm{if}\;u_{n}\in C_{k},\\ 0 & \mathrm{otherwise}. \end{array}\right.$$

Then, let set $I_{k}$ denote the index set of UAVs in cluster $C_{k}$, i.e., $I_{k}=\left\{n|x_{n,k}=1\right\}$.

Besides, let $y_{k,m}\in \left\{0,1\right\}$ denote whether cluster $C_{k}$ is assigned to localize NCT $g_{m}$, where

(2) $$y_{k,m}=\left\{\begin{array}{cc} 1 & \mathrm{if}\;C_{k}\to g_{m},\\ 0 & \mathrm{otherwise}. \end{array}\right.$$

### 3.2. Mobility-Aware Cluster Quality Metrics

Considering the mobility of UAVs and their changing positions, it is essential to maintain cluster nodes within an effective communication range and ensure similar movement trends. This contributes to network topology stability. Moreover, reducing communication overhead and delay within sensing clusters is crucial for preventing frequent topology changes. To achieve these objectives, we introduce three metrics to assess the cooperation quality of UAV clusters: position similarity, velocity similarity, and communication link consistency.

#### 3.2.1. Position Similarity and Velocity Similarity

For any two UAVs, their distance $d_{i,j}$ is measured by the Euclidean distance between their positions

(3) $$d_{i,j}={∥p_{i}-p_{j}∥}_{2}.$$

Next, we define the position similarity $D_{i,j}$ as follows.

**Definition** **1** (Position Similarity of a UAV Pair). *Position similarity $D_{i,j}$ of $u_{i}$ and $u_{j}$ is defined as*

(4) $$D_{i,j}=1-\frac{d_{i,j}}{d_{\max}},$$

*where $d_{\max}$ is a scale factor constant.*

Besides, we introduce the velocity similarity to evaluate the movement consistency of UAVs within a cluster.

**Definition** **2**  (Velocity Similarity of a UAV Pair). *Velocity similarity $V_{i,j}$ of $u_{i}$ and $u_{j}$ is defined as cosine similarity*

(5) $$V_{i,j}=\frac{v_{i}\cdot v_{j}}{∥v_{i}{∥}_{2}{∥v_{j}∥}_{2}}.$$

It can be observed that $D_{i,j}$ and $V_{i,j}$ are both symmetric, i.e., $D_{i,j}=D_{j,i}$ and $V_{i,j}=V_{j,i}$.

Next, we consider the metrics for clusters. To begin with, let $I_{k}^{\left(2\right)}$ denote the set of 2-element *unordered pairs* selected from the index set $I_{k}$ of $C_{k}$, as defined by Equation (6).

(6) Ik(2)=Ik2={(i,j)∣i,j∈Ik,i<j}.

To ensure the validity of the subsequent cluster metrics, we restrict our analysis to clusters with $|C_{k}|\geq 2$.

The cluster diameter is introduced to evaluate the spatial distribution of UAVs within a cluster.

**Definition 3**  (Cluster Diameter). *Cluster diameter $d_{k}$ is defined as the maximum distance between any UAV pairs within the cluster*

(7) $$d_{k}=\max_{\left(i,j\right)\in I_{k}^{\left(2\right)}}{∥p_{i}-p_{j}∥}_{2}.$$

**Definition 4**  (Position Similarity of a Cluster). *Position similarity of a cluster is the average position similarity of all UAV pairs within the cluster*

(8) $$D_{k}=\frac{1}{\left|I_{k}^{\left(2\right)}\right|}\sum_{\left(i,j\right)\in I_{k}^{\left(2\right)}}D_{i,j}.$$

**Remark 1.**  *When choosing $d_{\max}$ in Equation (4) to compute the position similarity of a UAV pair in the cluster, it is recommended to set $d_{\max}\geq \max_{\left(i,j\right)\in I_{k}^{\left(2\right)}}d_{i,j}$ to ensure it is within the normalized range [0,1].*

Similarly, the velocity similarity of a cluster can be defined as follows.

**Definition 5**  (Velocity Similarity of a Cluster). *Velocity similarity of a cluster is the average velocity similarity of all UAV pairs within the cluster*

(9) $$V_{k}=\frac{1}{\left|I_{k}^{\left(2\right)}\right|}\sum_{\left(i,j\right)\in I_{k}^{\left(2\right)}}V_{i,j}.$$

Finally, we define the motion similarity $\Phi_{k}$ of cluster $C_{k}$

**Definition 6**  (Motion Similarity of a Cluster). *Motion similarity $\Phi_{k}$ of cluster $C_{k}$ is defined as the weighted sum of position similarity and velocity similarity*

(10) $$\Phi_{k}=\epsilon D_{k}+\left(1-\epsilon \right)V_{k},$$

*where 0≤ϵ≤1 is a weight factor.*

#### 3.2.2. Communication Link Consistency

The communication link consistency [22] serves as a critical metric for evaluating information propagation performance in sensor networks. To ensure robust, rapid, and efficient intra-cluster communication among UAVs, we introduce a mobility-based model for communication link consistency.

**Figure 2:**
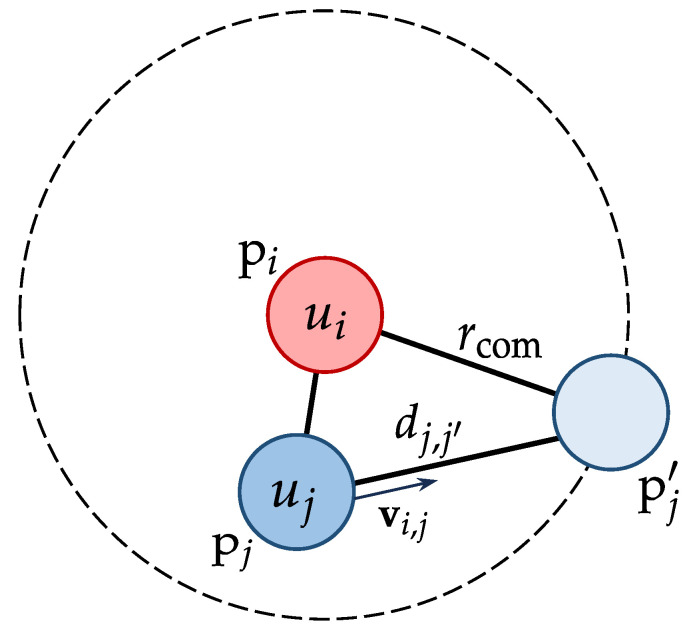
The positions and relative velocity of two UAVs $u_{i}$ and $u_{j}$ in the mobility-based model for defining communication link consistency $W_{i,j}$.

For two mobile UAVs $u_{i}$ and $u_{j}$ with identical communication radius $r_{\mathrm{com}}$, assuming constant relative velocity and direction over a short future time interval, the communication link consistency $W_{i,j}$ is defined as

(11) $$W_{i,j}=\left\{\begin{array}{cc} \frac{d_{j,j^{\prime }}}{∥v_{i,j}{∥}_{2}T_{\mathrm{th}}} & \mathrm{if}\;\frac{d_{j,j^{\prime }}}{∥v_{i,j}{∥}_{2}}<T_{\mathrm{th}},\\ 1 & \mathrm{if}\;\frac{d_{j,j^{\prime }}}{∥v_{i,j}{∥}_{2}}\geq T_{\mathrm{th}}, \end{array}\right.$$

where $v_{i,j}=v_{j}-v_{i}$ is the relative velocity, $p_{j}^{\prime }=p_{j}+\left(v_{i,j}/∥v_{i,j}{∥}_{2}\right)\cdot d_{j,j^{\prime }}$ is the projected position of $u_{j}$ relative to $u_{i}$ when the distance between them exactly reaches $r_{\mathrm{com}}$, $d_{j,j^{\prime }}\;=\;{∥p_{j}-p_{j}^{\prime }∥}_{2}$ is the Euclidean distance between $u_{j}$ and the projected position, and $T_{\mathrm{th}}$ is a time threshold for link stability, as illustrated in Figure 2.

Next, we define the communication link consistency of a cluster.

**Definition 7**  (Communication Link Consistency of a Cluster). *Communication link consistency $W_{k}$ of cluster $C_{k}$ is the average communication link consistency of all UAV pairs within the cluster*

(12) $$W_{k}=\frac{1}{\left|I_{k}^{\left(2\right)}\right|}\sum_{\left(i,j\right)\in I_{k}^{\left(2\right)}}W_{i,j}.$$

### 3.3. Cramér–Rao Lower Bound Derivation

The CRLB [5] is a fundamental benchmark in estimation theory, establishing a theoretical lower bound on the variance in any unbiased estimator. In the context of clustered UAV-based localization, the CRLB provides critical insights into the achievable localization accuracy under specific cluster configurations and measurement models. This section derives the CRLB for the proposed clustered localization framework, explicitly incorporating key factors such as the geometric distribution and velocity of UAVs within clusters, and the statistical properties of measurement errors.

The CRLB derivation builds upon the following fundamental assumptions.

- (A1) Measurement independence: Observations from different UAVs are statistically independent, i.e., $\delta_{m,n}$ is independent of $\delta_{m^{\prime },n^{\prime }}$ for $\left(m,n\right)\neq \left(m^{\prime },n^{\prime }\right)$.
- (A2) Error normality: Both position errors $\delta_{m,n}^{\mathrm{loc}}$ and motion-induced errors $\delta_{n}^{\mathrm{vel}}$ follow zero-mean Gaussian distributions.
- (A3) Spatial-temporal uncorrelation: Measurement errors across different time instants and spatial positions are mutually uncorrelated.

Using the TOA model [23], the real distance between a UAV located at $p_{n}$ and an NCT at $q_{m}$ is given by

(13) $$r_{m,n}={∥p_{n}-q_{m}∥}_{2}.$$

However, real-world observations from UAV $u_{n}$ for NCT $g_{m}$ are subject to normally distributed errors in sensor time-delay measurements, leading to noisy distance estimates

(14) $${\hat{r}}_{m,n}=r_{m,n}+e,$$

where *e* represents measurement noise. According to [24], the position error from distance measurement of NCTs follows a zero-mean normal distribution with variance, that is

(15) $$e∼N\left(0,\mu^{\mathrm{loc}}{\left(r_{m,n}\right)}^{4}\right),$$

where $\mu^{\mathrm{loc}}$ is a proportionality constant.

To transform this error into Euclidean coordinates, the estimated position ${\hat{q}}_{m,n}$ of $g_{m}$ observed by UAV *n* is given by

(16) $${\hat{q}}_{m,n}=q_{m}+\delta_{m,n}^{\mathrm{loc}},$$

where $\delta_{m,n}^{\mathrm{loc}}$ represents the equivalent localization noise, which follows a multivariate normal distribution

(17) $$\delta_{m,n}^{\mathrm{loc}}∼N\left(0,\Sigma_{m,n}^{\mathrm{loc}}\right).$$

where the covariance matrix $\Sigma_{m,n}^{\mathrm{loc}}$ can be computed as

(18) $$\Sigma_{m,n}^{\mathrm{loc}}=\mu^{\mathrm{loc}}{\left(r_{m,n}\right)}^{4}u_{m,n}u_{m,n}^{⊤},$$

where $u_{m,n}$ is the unit vector pointing from the UAV’s position $p_{n}$ to the NCT’s $q_{m}$.

Beyond position noise, UAV mobility introduces an additional source of error. The velocity $v_{n}$ of UAV *n* affects its self-localization accuracy, as positioning measurements from external sensors (e.g., GPS or infrared systems) experience time-delay errors. Assuming the UAV self-localizes at a fixed clock frequency, the positioning error tends to increase with its velocity magnitude $∥v_{n}{∥}_{2}$. This inherent localization uncertainty propagates to the position estimation of NCTs, particularly in high-speed scenarios. Consequently, the motion-induced localization error $\delta_{n}^{\mathrm{vel}}$ in Euclidean coordinates follows a multivariate normal distribution

(19) $$\delta_{n}^{\mathrm{vel}}∼N\left(0,\Sigma_{n}^{\mathrm{vel}}\right),$$

where $\Sigma_{n}^{\mathrm{vel}}$ is a velocity-dependent covariance matrix computed as

(20) $$\Sigma_{n}^{\mathrm{vel}}=\mu^{\mathrm{vel}}{∥v_{n}∥}^{2}d_{n}d_{n}^{⊤},$$

where $d_{n}=v_{n}/∥v_{n}{∥}_{2}$ is the unit velocity vector, and $\mu^{\mathrm{vel}}$ is a proportionality constant.

Combining these two sources of error, the total observed localization error for NCT *m* as measured by UAV $u_{n}$ is

(21) $$\delta_{m,n}=\delta_{m,n}^{\mathrm{loc}}+\delta_{n}^{\mathrm{vel}},$$

which, according to assumption (A2), follows a multivariate normal distribution,

(22) $$\delta_{m,n}∼N\left(0,\Sigma_{m,n}\right),$$

where the total covariance matrix is given by

(23) $$\Sigma_{m,n}=\Sigma_{m,n}^{\mathrm{loc}}+\Sigma_{n}^{\mathrm{vel}}.$$

**Figure 3:**
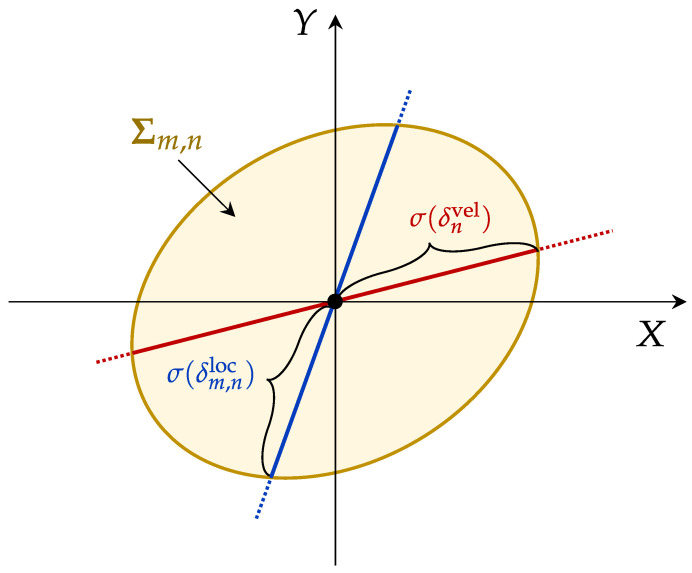
Illustration of the composite covariance matrix $\Sigma_{m,n}$ from two error components: position error $\delta_{n}^{\mathrm{loc}}$ and motion-induced error $\delta_{n}^{\mathrm{vel}}$ (denoted as $\Sigma_{n}^{\mathrm{loc}}$ and $\Sigma_{n}^{\mathrm{vel}}$ in Euclidean space, respectively). The red and blue lines represent error distributions, and σ(·) represents the standard deviation of the error.

Figure 3 illustrates the spatial characteristics and superposition effects of these error components. Note that the resulting composite covariance matrix $\Sigma_{m,n}$ may also be singular or ill-conditioned. To address this, we replace the standard matrix inverse with the Moore–Penrose pseudoinverse, denoted as $\Sigma_{m,n}^{+}$, ensuring that the inversion required for subsequent calculations (e.g., in the FIM and CRLB computations) remains well-defined.

Following conventional estimation theory [23,25,26], we then employ the CRLB to quantify localization precision. The FIM, denoted as J, serves as the fundamental construct for CRLB computation. For a single observation ${\hat{q}}_{m,n}$, the conditional probability density function (CPDF) is formulated as

(24) $$p\left({\hat{q}}_{m,n}|q_{m}\right)=\frac{1}{\sqrt{{\left(2\pi \right)}^{3}\left|\Sigma_{m,n}\right|}}\exp\left(-\frac{1}{2}{\left({\hat{q}}_{m,n}-q_{m}\right)}^{⊤}\Sigma_{m,n}^{+}\left({\hat{q}}_{m,n}-q_{m}\right)\right).$$

The corresponding log-likelihood function becomes

(25) $$L\left({\hat{q}}_{m,n}|q_{m}\right)=-\frac{1}{2}{\left({\hat{q}}_{m,n}-q_{m}\right)}^{⊤}\Sigma_{m,n}^{-1}\left({\hat{q}}_{m,n}-q_{m}\right)-\frac{1}{2}\log\left|\Sigma_{m,n}\right|-\frac{3}{2}\log\left(2\pi \right).$$

For a UAV cluster $C_{k}$ with the UAV index set $I_{k}$ assigned to localize NCT $g_{m}$, the joint CPDF under Assumptions (A1) and (A3) is

(26) $$p\left({\hat{q}}_{m,k}|q_{m}\right)=\prod_{n\in I_{k}}p\left({\hat{q}}_{m,n}|q_{m}\right).$$

Then, the aggregate log-likelihood function consequently follows

(27) $$L\left({\hat{q}}_{m,k}|q_{m}\right)=\sum_{n\in I_{k}}L\left({\hat{q}}_{m,n}|q_{m}\right)=\sum_{n\in I_{k}}\log p\left({\hat{q}}_{m,n}|q_{m}\right).$$

In 3D Euclidean space, the FIM adopts the symmetric structure

(28) $$J=\left[\begin{array}{ccc} J_{XX} & J_{XY} & J_{XZ}\\ J_{XY} & J_{YY} & J_{YZ}\\ J_{XZ} & J_{YZ} & J_{ZZ} \end{array}\right],$$

where each element is computed by

(29) $$J_{\theta_{1}\theta_{2}}\left({\hat{q}}_{m,k}\right)=-E\left[\frac{\partial^{2}L\left({\hat{q}}_{m,k}\right)}{\partial \theta_{1}\partial \theta_{2}}\right],$$

where $\theta_{1},\theta_{2}\in \left\{X,Y,Z\right\}$. Leveraging multivariate normal distribution properties, the FIM simplifies to

(30) $$J=\sum_{n\in I_{k}}\Sigma_{m,n}^{+}.$$

The additive structure of FIM $J=\sum_{n\in I_{k}}\Sigma_{m,n}^{+}$ directly results from the assumption (A1), as cross-correlation terms between different UAV observations vanish.

Then, the CRLB $B_{k,m}$ of NCT *m* for the configured cluster $C_{k}$ is thereby computed as

(31) $$B_{k,m}=\mathrm{CRLB}\left(C_{k},m\right)=\mathrm{tr}\left(J^{+}\right)=\mathrm{tr}\left({\left(\sum_{n\in I_{k}}\Sigma_{m,n}^{+}\right)}^{+}\right),$$

where tr(·) denotes matrix trace and ${\left(\cdot \right)}^{+}$ is the Moore–Penrose pseudoinverse of $\left(\cdot \right)$.

### 3.4. Problem Formulation for Optimal Cluster Assignment

We first consider the constraints among UAVs, clusters, and targets. Recall that *x* and *y* are indicator variables defined by Equations (1) and (2). Here, we assume each UAV can only be assigned to one cluster (The framework can be generalized by allowing each UAV to participate in multiple clusters, defined by $\sum_{k=1}^{K}x_{n,k}\leq \kappa_{n}$, where $\kappa_{n}$ specifies the maximum number of clusters UAV $u_{n}$ can belong to. This extension maintains mathematical consistency with our model. For simplicity, we focus on the case that $\kappa_{n}\equiv 1$ in this paper.), i.e., $\sum_{k=1}^{K}x_{n,k}\leq 1,\forall n\in \left\{1,\ldots ,N\right\}$, while each cluster corresponds to one NCT, i.e., $\sum_{m=1}^{M}y_{k,m}=1$ and $\sum_{k=1}^{K}y_{k,m}=1$, ∀k∈{1,…,K},∀m∈{1,…,M}. Besides, each cluster must have at least *c* UAVs, i.e., $\sum_{n=1}^{N}x_{n,k}\geq c,\forall k\in \left\{1,\ldots ,K\right\}$, which also implies that the total number of UAVs must be at least *c* times the number of NCTs. Additionally, each cluster has a maximum radius constraint, i.e., $d_{k}\leq d_{\mathrm{th}},\forall k\in \left\{1,\ldots ,K\right\}$.

Next, we define the objective function. For an NCT $g_{m}$, both motion similarity $\Phi_{k(m)}$ and communication link consistency $W_{k(m)}$ of its corresponding cluster $C_{k(m)}$ should be maximized to ensure effective collaboration while its CRLM $B_{k(m),m}$ should be minimized to ensure localization performance. Thus, the objective function considering all NCTs and their corresponding clusters is formulated as

(32) $$\min\max_{m}\left(\alpha_{1}B_{k(m),m}-\alpha_{2}\Phi_{k(m)}-\alpha_{3}W_{k(m)}\right),$$

where k(m) indicates that *k* is the index of the cluster assigned to the NCT $g_{m}$ decided by *y*, and $\alpha_{1},\alpha_{2},\alpha_{3}$ are weight factors.

Thus, the optimization problem is formulated as P1.

(33) $$\begin{array}{ccc} P1:\;\min_{x,y} & \;\max_{m}\left(\alpha_{1}B_{k,m(k)}-\alpha_{2}\Phi_{m(k)}-\alpha_{3}W_{m(k)}\right)\\ s.t. & \;\sum_{k=1}^{K}x_{n,k}\leq 1,\;\forall n\in \left\{1,\ldots ,N\right\},\; & \left(C1\right)\\ \; & \;\sum_{m=1}^{M}y_{k,m(k)}=1,\;\forall k=\left\{1,\ldots ,K\right\},\; & \left(C2\right)\\ \; & \;\sum_{k=1}^{K}y_{k,m(k)}=1,\;\forall m\in \left\{1,\ldots ,M\right\}, & \;\left(C3\right)\\ \; & \;\sum_{n=1}^{N}x_{n,m(k)}\geq c,\;\forall k\in \left\{1,\ldots ,K\right\}, & \;\left(C4\right)\\ \; & \;d_{k}\leq d_{\mathrm{th}},\;\forall k\in \left\{1,\ldots ,K\right\}.\; & \left(C5\right) \end{array}$$

The formulated optimization problem is a zero-one programming problem, where the decision variables *x* and *y* are binary integers indicating the assignment of UAVs to clusters and the corresponding relationship between NCTs and clusters.

The formulated optimization problem P1 is computationally challenging due to multiple interrelated factors. First, the objective function involves nonlinear matrix operations through the CRLB term $B_{k,m(k)}=\mathrm{tr}{\left(\sum_{n\in I_{k}}\Sigma_{m,n}^{+}\right)}^{+}$. These operations include nested matrix inversions and trace computations, leading to a non-convex structure with many local minima. Second, the discrete constraints (C1)–(C4) introduce an NP-hard combinatorial structure.

While the original formulation P1 captures the full complexity of dynamic UAV clustering, its direct solution remains computationally intractable due to high-dimensional combinatorial search. Given the constraints (C2) and (C3) in the optimization problem P1, the variable *y* constitutes a permutation matrix, which inherently implies that the equality K=M must hold. This relationship leads to two significant consequences: First, k(m) becomes a single-valued function, allowing to use $B_{k(m)}$ to represent $B_{k,m(k)}$ for simplicity. Second, the correspondence between *m* and *k* establishes a bijective mapping. Consequently, each cluster $C_{k}$ can be uniquely associated with a certain NCT m(k), enabling direct parameterization through either cluster indices {1,…,K} or NCT indices {1,…,M}. In this paper, we adopt the cluster index notation. Thus, the reformulated problem becomes P2.

(34) $$\begin{array}{ccc} P2:\;\min_{x} & \;\max_{k}\left(\alpha_{1}B_{k}-\alpha_{2}\Phi_{k}-\alpha_{3}W_{k}\right)\\ s.t. & \;\sum_{k=1}^{K}x_{n,k}\leq 1,\;\forall n\in \left\{1,\ldots ,N\right\},\; & \left(C1\right)\\ \; & \;\sum_{n=1}^{N}x_{n,k}\geq c,\;\forall k\in \left\{1,\ldots ,K\right\}, & \;\left(C4\right)\\ \; & \;d_{k}\leq d_{\mathrm{th}},\;\forall k\in \left\{1,\ldots ,K\right\}.\; & \left(C5\right) \end{array}$$

The reformulated P2 is also a zero-one programming problem, where the decision variables *x* are binary integers. Even after simplification, P2 retains its non-convex and non-linear nature, making it challenging to solve. Therefore, it is necessary to establish an efficient algorithm to find a solution within a reasonable amount of computational time.

## 4. Methodology

### 4.1. Algorithm Framework

**Figure 4:**
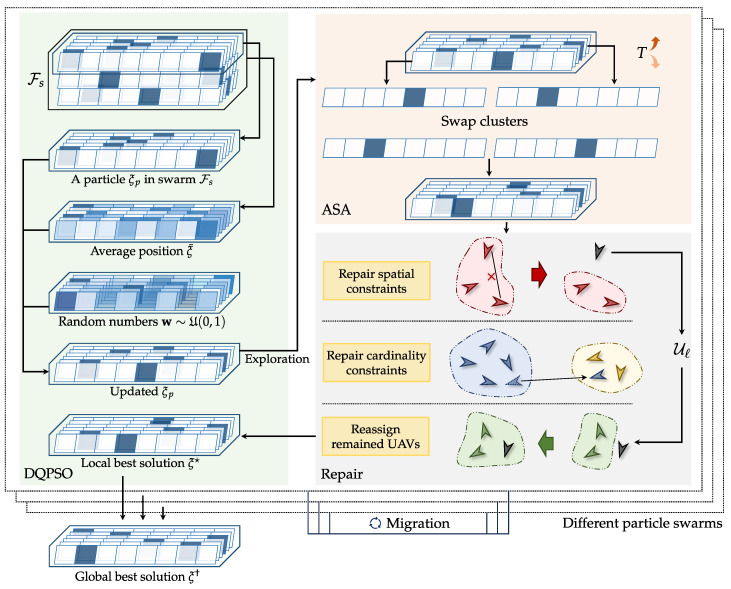
Framework of the proposed MDQPSO-ASA algorithm, which integrates multi-swarm optimization, discrete quantum-inspired PSO, adaptive simulated annealing, and a repair mechanism for solutions.

In this section, we present the proposed Multi-swarm Discrete Quantum-inspired Particle Swarm Optimization with Adaptive Simulated Annealing (MDQPSO-ASA) algorithm, a novel computational framework specifically designed to resolve the NP-hard dynamic UAV clustering problem. As depicted in Figure 4, MDQPSO-ASA integrates three synergistic components through bio-inspired computing principles: a multi-swarm optimization framework, a discrete quantum-inspired PSO module (DQPSO), and an adaptive simulated annealing (ASA) module. The multi-swarm framework enhances the diversity of the search process, increasing the global search capability and preventing premature convergence. The quantum-inspired updates help the algorithm escape local optima and improve the overall search effectiveness. The ASA module enables the algorithm to navigate through different areas of the solution space. Overall, the discrete nature of MDQPSO-ASA is particularly suitable for the zero-one integer programming problem presented in this paper, ensuring that the solutions adhere to the binary constraints. Additionally, a repair mechanism is incorporated to attempt to fix solutions that do not satisfy the cluster radius constraints. These features make MDQPSO-ASA well-suited for the dynamic and complex UAV clustering and multi-target localization scenarios addressed in this work. The overall procedure is detailed in Algorithm 1, and each component is explained in the following subsections based on its corresponding pseudocode.

In Algorithm 1, the multi-swarm framework initializes multiple swarms ${\left\{F_{s}\right\}}_{s=1}^{S}$ with random strategies (Line 1), ensuring a diverse set of starting points. Each swarm consists of *P* particles, whose positions ${\left\{\xi_{s}\right\}}_{s=1}^{S}$ representing potential solutions to the optimization problem. The temperature *T* and cooling rate *r* are initialized (Line 2) to control the simulated annealing process. The temperature adaptation counter is also initialized to track the progress of the temperature adaptation mechanism. The algorithm then initializes the global best $\xi^{†}$ and local best $\xi_{s}^{★}$ for each swarm (Line 3), which are updated throughout the optimization process, allowing the algorithm to adaptively guide the search. The outer loop (Line 5) iterates until the maximum iteration count $it_{\max}$ is reached. For each iteration, a counter is incremented to track progress for temperature adaptation.

Then, for each swarm $F_{s}$ (Line 7), the DQPSO module is applied (Line 8) to update the positions of all particles using discrete quantum-inspired dynamics. Every $\tau_{1}$ iterations (Line 9), the ASA module (Line 10) perturbs the current solutions based on a simulated annealing mechanism to escape local minima. Then, a repair mechanism (see Section 4.4) is adopted (Line 12) to ensure the feasibility of the solution. After processing the particles, a migration operation (Line 20) exchanges information among different swarms; that is, at fixed intervals $\tau_{2}$, the migration operation compares the best local solutions ${\left\{\xi_{s}^{★}\right\}}_{s=1}^{S}$ within each swarm and migrates better solutions from swarms to swarms. This facilitates information exchange across the population and enhances global search capability.

**Algorithm 1** Framework of the proposed algorithm MDQPSO-ASA.
**Require:** 
Cost function C(ξ), acceleration coefficient β, number of swarms *S*, number of particles per swarm *P*, initial temperature $T_{0}$, cooling rate $r_{0}$, ASA interval $\tau_{1}$, immigration interval $\tau_{2}$**Ensure:** 
Global optimum $\xi^{†}$, global minimum cost $C_{\min}$1:Initialize a set of swarms $F={⋃}_{s=1}^{S}\left\{F_{s}\right\}$ with random strategy where $F_{s}={⋃}_{p=1}^{P}\left\{\xi_{p}\right\}$2:Set initial temperature $T\leftarrow T_{0}$ and initial cooling rate $r\leftarrow r_{0}$3:Initialize global best $\xi^{†}$ of F and local best $\xi_{s}^{★}$ of each $F_{s}$4:Initialize temperature adaptation counter cnt←05:
**for** $\;it=0,1,\ldots ,it_{\max}-1\;$ **do**
6:      cnt←cnt+17:     **for** each swarm $F_{s}\in F$ **do**8:           $F_{s}\leftarrow$DQPSO($F_{s},\beta$)                         ▷ see Section 4.2 and Algorithm 29:           **if** $it\;\mathrm{mod}\;\tau_{1}=0$ **then**10:                $F_{s}\leftarrow$SA($F_{s},T$)                          ▷ see Section 4.3 and Algorithm 311:        **end if**12:        $F_{s}\leftarrow$Repair($F_{s}$)                               ▷ see Section 4.4 and Algorithm 413:        **for** each particle $\xi_{p}$ in $F_{s}$ **do**14:              **if** $C\left(\xi_{p}\right)<C\left(\xi_{s}^{★}\right)$ **then**15:                   Update local best $\xi_{s}^{★}\leftarrow \xi_{p}$16:              **end if**17:        **end for**18:    **end for**19:    **if** $it\;\mathrm{mod}\;\tau_{2}=0$ **then**20:        F←Migration(F)21:    **end if**22:    **for** each local best $\xi_{s}^{★}$ of swarm $F_{s}$ in F **do**23:        **if** $C\left(\xi_{s}^{★}\right)<C\left(\xi^{†}\right)$ **then**24:              Update global best $\xi^{†}\leftarrow \xi_{s}^{★}$25:              Reset counter cnt←026:        **end if**27:    **end for**28:    T,r,cnt←TempAdapt(T,r,it,cnt)                                  ▷ see Section 4.329:
**end for**



Finally, the algorithm checks each swarm’s best solution and updates the global best $\xi^{†}$ if a better solution is found, while also resetting the temperature adaptation counter (Line 24). Next, the temperature *T* is updated using an adaptation mechanism (Line 28) that takes into account the cooling rate *r* and the stagnation counter cnt. This layered structure helps balance exploration and exploitation throughout the iterative process.

Note that in the algorithms introduced in this section, notation ξ represents the position of a particle in PSO, which corresponds to but is not identical to the decision variable *x*. The position ξ consists of continuous variables used in the optimization processes to represent potential solutions, while *x* consists of binary decision variables indicating the assignment of UAVs to clusters. The optimization process involves discretizing ξ to obtain feasible values for *x*, which will be further introduced in the DQPSO module (Section 4.2).

### 4.2. Discrete Quantum-Inspired PSO (DQPSO) Module

The DQPSO module, which is described in detail as Algorithm 2, is responsible for updating the positions of particles within each swarm by incorporating quantum-inspired principles.

**Algorithm 2** DQPSO module.
**Require:** 
Swarm $F_{s}$ and acceleration coefficient β**Ensure:** 
Updated swarm $F_{s}$1:Compute mean position $\bar{\xi }\leftarrow \frac{1}{P}\sum_{p=1}^{P}\xi_{p}$2:**for** each particle $\xi_{p}$ in $F_{s}$ **do**3:    Compute quantum rotation magnitude $Q_{p}\leftarrow \beta \cdot {∥\bar{\xi }-\xi_{p}∥}_{2}$4:    Generate a set of random numbers w∼U(0,1)5:    Update position $\xi_{p}\leftarrow \xi_{p}+Q_{p}⊙\ln\left(1/w\right)$6:    Discretize $\xi_{p}\leftarrow$Argmax–Softmax($\xi_{p}$)7:
**end for**



Initially, the mean position $\bar{\xi }$ of all particles is computed (Line 1). Then, for each particle, the quantum rotation magnitude $Q_{p}$ is calculated as the product of the acceleration coefficient β and the Euclidean distance between the particle’s current position and the mean position (Line 3). A set of random numbers w with the same shape as ξ drawn from a uniform distribution in U(0,1) is used to generate a logarithmic perturbation, which is then added to the current position (Line 5). Since the optimization problem is discrete, the updated continuous value is discretized using a softmax-argmax combined function to compute the probability of the UAV switching to each cluster (Line 6). The cluster with the highest probability is then selected using the argmax function, setting its value to 1 and all others to 0. This ensures that the new positions adhere to the binary constraints of the problem.

### 4.3. Adaptive Simulated Annealing (ASA) Module

The ASA module, which consists of the temperature adaptation process and the SA procedure, ensures that the algorithm can traverse different regions of the solution space. For the temperature adaptation process, given the variables *T*, *r*, it and cnt, the new *r* and temperature *T* can be updated by Equations (35) and (36), respectively.

(35) r←0.9·r,cnt←0ifcnt≥5,

(36) T←T·exp(−r·it).

The adaptive nature of the temperature parameter *T*, updated externally in the main framework, regulates the acceptance probability, balancing between exploration (high temperature) and exploitation (low temperature).

The SA procedure, as shown in Algorithm 3, is the key to the ASA module, which is designed to introduce thermal perturbations to each particle in the swarm, which helps in escaping local optima. For every particle, a neighboring solution $\xi_{i}^{\prime }$ is generated by applying a perturbation function (Line 2), which randomly selects two UAVs and swaps their cluster assignments. The energy difference ΔE, calculated as the difference between the cost of the new and current solutions, quantifies the change in objective value (Line 3). Using a randomly generated number *h* from a uniform distribution U(0,1) and the Metropolis acceptance criterion, the algorithm accepts the new solution if it yields a lower cost or with a probability proportional to exp(−ΔE/T) when the cost is higher (Lines 5 and 6). This controlled acceptance mechanism allows the swarm to occasionally accept worse solutions, thereby maintaining diversity and improving the potential for global exploration.

**Algorithm 3** SA procedure.
**Require:** 
Swarm $F_{s}$, temperature *T***Ensure:** 
Modified swarm $F_{s}$1:**for** each particle $\xi_{p}$ in $F_{s}$ **do**2:    Generate neighbor $\xi_{p}^{\prime }\leftarrow$Perturb($\xi_{p}$)3:    Compute energy difference $\Delta E\leftarrow C\left(\xi_{p}^{\prime }\right)-C\left(\xi_{p}\right)$4:    Generate a random number h∼U(0,1)5:    **if** ΔE<0 or exp(−ΔE/T)>h **then**6:          $\xi_{p}\leftarrow \xi_{p}^{\prime }$7:    **end if**8:
**end for**



### 4.4. Repair Mechanism

The repair mechanism ensures solution feasibility by enforcing both spatial and cardinality constraints through a three-stage process, as formalized in Algorithm 4. This critical component guarantees that all candidate solutions satisfy conditions (C4) and (C5).

(37) $$\begin{array}{ccc} \; & |C_{k}|\geq c,\;\forall k\in \left\{1,\ldots ,K\right\} & \;\left(C4\right)\\ \; & \max_{\left(i,j\right)\in I_{k}^{\left(2\right)}}{∥p_{i}-p_{j}∥}_{2}\leq d_{\mathrm{th}}\;\forall k\in \left\{1,\ldots ,K\right\} & \;\left(C5\right) \end{array}$$

In the first stage, the algorithm enforces the spatial constraints within each cluster. For every cluster $C_{k}$, it computes the centroid $\chi_{k}$ (Line 4) and then identifies the pair of UAVs that are farthest apart (Line 5). If the distance between these two UAVs exceeds the threshold $d_{\mathrm{th}}$ (Line 6), the UAV farther from the centroid within the pair is removed from the cluster and placed into a temporary set $U_{\ell }$ (Lines 7 and 8). This process is repeated until the maximum pairwise distance within the cluster is within the allowable range, ensuring spatial compactness and communicational efficiency.

The second stage addresses the cardinality constraints. For any cluster $C_{k}$ with fewer than *c* members (Line 12), the algorithm creates a candidate set V by gathering UAVs from other clusters that exceed the minimum size (Line 13). These candidate UAVs are randomly shuffled (Line 14), and each candidate is evaluated to check if its addition to the under-populated cluster will keep the spatial constraint satisfied (Line 15). When a candidate UAV meets the condition, it is moved from its original cluster $C_{k^{\prime }}$ to the deficient one $C_{k}$ (Line 16). This reallocation continues until the cluster achieves the minimum required cardinality.

In the third stage, the algorithm attempts to reassign any UAVs that remain unassigned in the set $U_{\ell }$. Each UAV in this set is considered for reassignment to the cluster whose centroid is closest, provided that its inclusion does not violate the spatial constraint (Line 22). If such a cluster is found, the UAV is added to that cluster and removed from $U_{\ell }$ (Lines 23 and 24). Finally, the algorithm checks whether every cluster meets the cardinality constraint. If any cluster still has fewer than *c* UAVs, the repair is deemed unsuccessful (Line 27); otherwise, the repaired solution $\xi_{i}^{\prime }$ is reconstructed from the adjusted clusters (Line 28).

This three-stage process systematically adjusts clusters to ensure both spatial compactness and sufficient cardinality, thereby maintaining feasibility for the subsequent optimization process.

**Algorithm 4** Repair mechanism
**Require:** 
Invalid solution $\xi_{i}$**Ensure:** 
Repaired solution $\xi_{i}^{\prime }$1:Initialize $U_{\ell }\leftarrow \emptyset$2:**for** each cluster $C_{k},\;k\in \left\{1,\ldots ,K\right\}$**do**                                             ▷ first stage3:      **repeat**4:            Compute cluster centroid $\chi_{k}\leftarrow \frac{1}{|C_{k}|}\sum_{u_{n}\in C_{k}}p_{n}$5:            $\left(u_{i},u_{j}\right)\leftarrow \arg\max_{\left(i,j\right)\in I_{k}^{\left(2\right)}}{∥p_{i}-p_{j}∥}_{2}$6:            **if** $∥p_{i}-p_{j}{∥}_{2}>d_{\mathrm{th}}$ **then**7:                 $u_{*}\leftarrow \arg\max_{u_{*}\in \left\{u_{i},u_{j}\right\}}{∥p_{*}-\chi_{k}∥}_{2}$8:                 $C_{k}\leftarrow C_{k}\setminus \left\{u_{*}\right\},\;U_{\ell }\leftarrow U_{\ell }\cup \left\{u_{*}\right\}$9:            **end if**10:    **until** $∥p_{i}-p_{j}{∥}_{2}\leq d_{\mathrm{th}}$11:
**end for**
12:**for** each cluster $C_{k}$ with $|C_{k}|<c$**do**                                         ▷ second stage13:       $V\leftarrow {⋃}_{k^{\prime }\neq k}\{u_{n}\in C_{k^{\prime }}||C_{k^{\prime }}\left|>c\right\}$14:       **for** $u_{n}\in$Shuffle(V) (also, $u_{n}\in C_{k^{\prime }}$) **do**15:              **if** $\max_{i\in I_{k}}{∥p_{n}-p_{i}∥}_{2}\leq d_{\mathrm{th}}$**and**$|C_{k^{\prime }}|>c$ **then**16:                      $C_{k}\leftarrow C_{k}\cup \left\{u_{n}\right\},\;C_{k^{\prime }}\leftarrow C_{k^{\prime }}\setminus \left\{u_{n}\right\}$17:                      **break if** $|C_{k}|\geq c$18:              **end if**19:        **end for**20:
**end for**
21:**for** $u_{n}\in$Shuffle($U_{\ell }$) **do**                                                             ▷ third stage22:      Try to find $C_{k}$ minimizing $∥p_{n}-\chi_{k}{∥}_{2}$ subject to $\max_{i\in I_{k}}{∥p_{n}-p_{i}∥}_{2}\leq d_{\mathrm{th}}$23:      **if** such $C_{k}$ exists **then**24:        $C_{k}\leftarrow C_{k}\cup \left\{u_{n}\right\},\;U_{\ell }\leftarrow U_{\ell }\setminus \left\{u_{n}\right\}$25:      **end if**26:
**end for**
27:**return** $\xi_{i}$ **if** $\exists \;C_{k}$ such that $|C_{k}|<c$                                          ▷ repair failed28:**return** $\xi_{i}^{\prime }$ reconstructed from ${\left\{C_{k}\right\}}_{k=1}^{K}$                                ▷ repair succeed


## 5. Performance Evaluation

### 5.1. Experiment Setup

In this section, we evaluate the performance of the proposed algorithm under a typical scenario where UAVs and NCTs are randomly distributed within a 3D region. We investigate the impact of varying the number of UAVs and NCTs on the algorithm’s performance, measured by the objective function in P2, and assess the algorithm’s runtime. All experiments are executed on a Windows 11 laptop with an Intel Core i9-12900H CPU and 32 GB RAM.

The UAVs and NCTs are randomly and uniformly distributed within a 1000×1000×1000 3D environment. Table 1 presents the default parameter settings for the simulation experiments.

### 5.2. Baseline Algorithms

We compare the proposed MDQPSO-ASA algorithm with several baseline algorithms to evaluate its performance in dynamic UAV clustering and multi-target localization. The algorithms in the comparison include the following algorithms:

- Simulated annealing (SA): A classical optimization algorithm that simulates the annealing process [28] to escape local optima.
- SA with genetic algorithm (SA-GA): A hybrid optimization algorithm that combines simulated annealing with genetic algorithm [29] to enhance the search capability.
- Discrete PSO (DPSO): A discrete version of PSO that enforces binary constraints [30] on the particle positions.
- Quantum-inspired PSO (QPSO): A quantum-inspired optimization algorithm that uses quantum rotation gates [31] to update particle positions.
- MDQPSO: The simplified MDQPSO-ASA algorithm without the ASA module.

In subsequent experiments, we test each algorithm in different cases 100 times and then take the average values along with the variances for performance evaluation.

### 5.3. Experiment Results and Analysis

#### 5.3.1. Convergence Analysis

**Figure 5:**
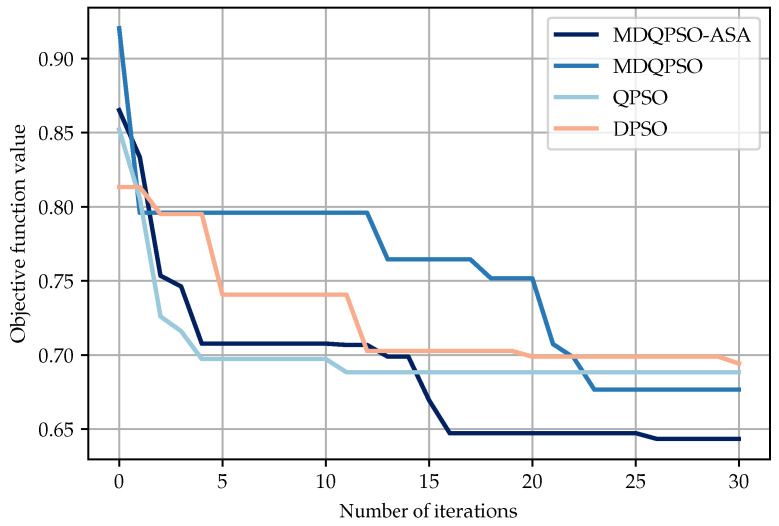
Curves for objective functions of different algorithms over 30 iterations.

To evaluate the convergence properties of the proposed MDQPSO-ASA algorithm, we compare its performance with several baseline optimization algorithms, including MDQPSO, QPSO, and DPSO. Figure 5 illustrates the convergence curves of these algorithms over 30 iterations. In the first five iterations, MDQPSO-ASA reduces the objective slightly slower than QPSO. Between iterations 5 and 10, both curves of QPSO and MDQPSO-ASA stabilize, yet QPSO remains 1.45 % better than MDQPSO-ASA. From iterations 10 to 20, MDQPSO-ASA resumes lowering the objective, finding better solutions, while QPSO stops improving. In the final stage (iterations 20–30), MDQPSO-ASA continues to refine its solution and achieves the lowest objective value among all methods. In conclusion, MDQPSO-ASA establishes a more balanced exploration-exploitation trade-off through its adaptive mechanisms, leading to enhanced long-term stability and significantly lower final objective values. This improved convergence performance highlights the algorithm’s effectiveness in overcoming local optima and identifying high-quality solutions through dynamic strategy adjustments. Although we did not compare SA-based algorithms directly due to the inherent differences in their iteration structures, our subsequent experiments will include a comparative analysis of PSO-based and SA-based algorithms to provide a comprehensive performance evaluation.

#### 5.3.2. Clustering Results for Different Scales

**Figure 6:**
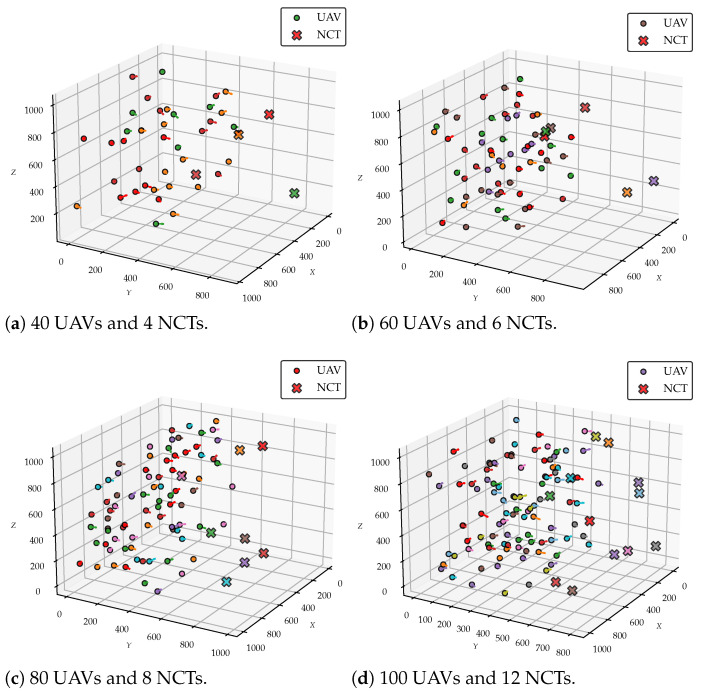
Clustering results for different scales of UAVs and NCTs.

To illustrate the effectiveness of the proposed clustering algorithm, we present the clustering results for various scales of UAVs and NCTs. Figure 6 includes subfigures (a)–(d), each depicting different configurations with clusters color-coded for clarity, UAVs with the same color belong to the same cluster and collaboratively localize the NCT of the corresponding color: (a) 40 UAVs and 4 NCTs, (b) 60 UAVs and 6 NCTs, (c) 80 UAVs and 8 NCTs, and (d) 100 UAVs and 12 NCTs. These results demonstrate that the MDQPSO-ASA algorithm can efficiently form clusters that adhere to spatial and cardinality constraints, ensuring effective collaboration among UAVs for multi-target localization.

#### 5.3.3. Solution Quality for Different Numbers of UAVs and NCTs

**Figure 7:**
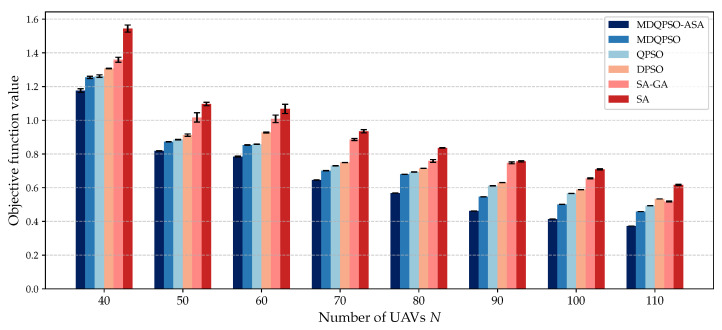
Solution quality for different numbers of UAVs compared to baseline algorithms with M=8 NCTs.

**Figure 8:**
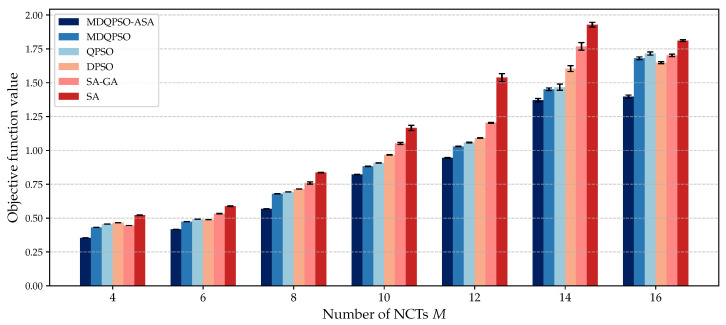
Solution quality for different numbers of NCTs compared to baseline algorithms with N=80 UAVs.

We compare the solution quality of different algorithms under different numbers of UAVs and NCTs. Figure 7 and Figure 8 illustrate the objective values obtained by each algorithm for different configurations.

Figure 7 presents the solution quality of various algorithms for different numbers of UAVs (N=40,50,…,110) while maintaining a fixed number of NCTs at M=8. It is evident that in each configuration, the proposed MDQPSO-ASA method consistently achieves the lowest objective values, indicating its ability to find better feasible solutions compared to other algorithms. This demonstrates the superior solution quality of MDQPSO-ASA. Additionally, the performance hierarchy among the algorithms remains consistent across different scales, with MDQPSO-ASA performing the best and SA performing the worst. Furthermore, the experiments indicate that the ASA module enhances the solution quality of MDQPSO by comparing the performance between MDQPSO and MDQPSO-ASA. As the number of UAVs increases, the objective values of all algorithms tend to decrease, which is expected since more UAVs provide better coverage and collaboration opportunities for localization tasks. However, the rate of improvement varies among the algorithms. MDQPSO-ASA shows a more significant reduction in objective values compared to other algorithms, highlighting its effectiveness in leveraging additional UAVs for improved localization accuracy. Moreover, the stability of MDQPSO-ASA is evident from the relatively smaller variance in objective values across different runs, suggesting that the algorithm consistently finds high-quality solutions. In contrast, other algorithms exhibit larger variances, indicating less reliable performance. This robustness is particularly important in dynamic and uncertain environments where consistent performance is crucial.

Figure 8 illustrates the solution quality of different algorithms for different numbers of NCTs (M=4,6,…,16) while keeping the number of UAVs fixed at N=80. MDQPSO-ASA consistently achieves the lowest objective values, demonstrating its superior performance in finding high-quality solutions. As the number of NCTs increases, the objective values of all algorithms rise, which is expected due to the increased complexity of clustering and localization. However, MDQPSO-ASA shows a more gradual increase, highlighting its robustness and adaptability. The results also indicate that MDQPSO-ASA maintains a smaller variance in objective values across different runs, suggesting stable and reliable performance. In contrast, other algorithms exhibit larger variances, indicating less consistent performance.

Overall, MDQPSO-ASA outperforms other algorithms in solution quality and maintains stable performance across different scales of UAVs or NCTs, making it an efficient algorithm for dynamic UAV clustering and multi-target localization.

#### 5.3.4. Solution Quality for Different Key Threshold Parameters

**Figure 9:**
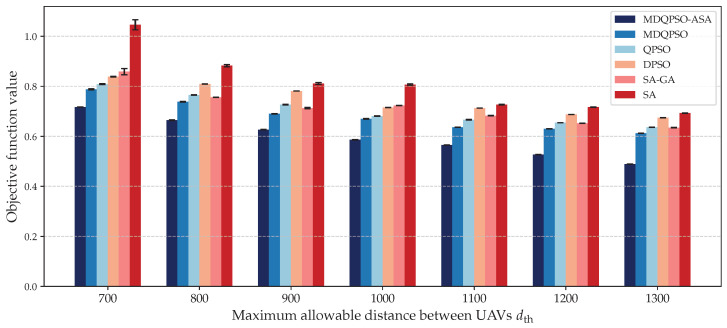
Solution quality for different maximum allowable distances between UAVs in a cluster compared to baseline algorithms.

**Figure 10:**
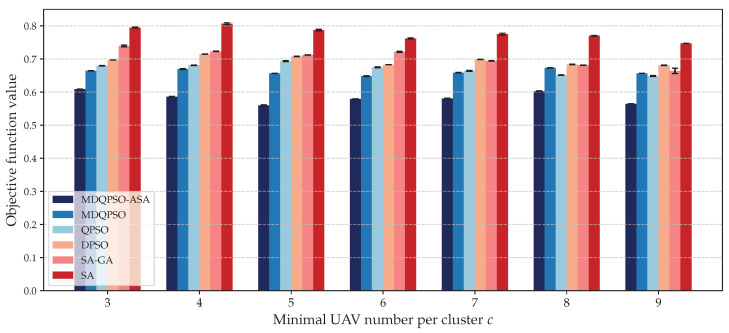
Solution quality for different minimal UAV numbers per cluster compared to baseline algorithms.

We compare the solution quality of different algorithms under different key threshold parameters: (1) maximum allowable distance between UAVs in a cluster $d_{\mathrm{th}}$, and (2) minimal UAV number per cluster *c*. Figure 9 and Figure 10 illustrate the objective values obtained by each algorithm for different configurations.

First, to evaluate the robustness of the proposed algorithm under different clustering constraints, we conducted a sensitivity analysis by varying the maximum allowable distance between UAVs in a cluster $d_{\mathrm{th}}$ from 700 to 1300. We recorded the mean value and the variance in the resulting objective function values. Figure 9 presents the solution quality of each algorithm for different settings of maximum allowable distance while all other parameters remain constant. As $d_{\mathrm{th}}$ increases, the cluster formation becomes less spatially constrained, which generally allows more flexible UAV grouping. This leads to a gradual decrease in objective values for most algorithms, and the performance improvements vary across methods. Throughout the entire range of $d_{\mathrm{th}}$, MDQPSO-ASA consistently achieves the lowest mean objective values, indicating the highest solution quality. Moreover, its variance remains minimal, demonstrating strong stability and robustness to changes in spatial constraints. These results validate that MDQPSO-ASA not only adapts well to different spatial constraint settings but also provides superior and reliable performance with different $d_{\mathrm{th}}$ settings.

Second, to further evaluate the robustness of the proposed algorithm, we conducted a sensitivity analysis with respect to the parameter *c* of minimal UAV number per cluster *c*, which defines the minimal UAV number per cluster. We varied *c* from 3 to 9 and recorded the mean value and the variance in the resulting objective function values. Figure 10 presents the solution quality of each algorithm for different settings of minimal UAV number per cluster, while all other parameters remain constant. As *c* increases, the algorithm faces stricter constraints in forming feasible clusters, which may reduce clustering flexibility and increase task difficulty. Meanwhile, it may also lead to an increase in the frequency of triggering the repair process and a decrease in the probability of successful repair. Across different values of *c*, MDQPSO-ASA consistently achieves the lowest mean objective function values, demonstrating its superior optimization ability under varying constraint levels. Notably, it maintains very low variance, ensuring consistent performance and high solution stability even when the system is forced to form larger clusters under stricter constraints. This indicates that the proposed algorithm not only achieves high-quality solutions on average but also exhibits strong reliability, which is critical for practical multi-target localization tasks where consistent outcomes are essential. These results confirm that MDQPSO-ASA is not only effective under normal conditions but also exhibits strong robustness when facing more stringent task constraints, such as requiring more UAVs per cluster. This robustness makes it particularly suitable for real-world multi-target localization missions where minimum resource guarantees per task are critical.

#### 5.3.5. Evaluation of Repair Mechanism

The repair mechanism is an essential part of the proposed MDQPSO-ASA algorithm, so in this section, we provide the quantitative evaluation of this mechanism. Specifically, we consider the following two indicators

- Triggering frequency of repair operation during an iteration of the optimization process, as denoted by $f_{r}$.
- Success rate of repair operation $s_{r}$, i.e., the proportion of infeasible solutions that are successfully repaired into feasible ones.

**Figure 11:**
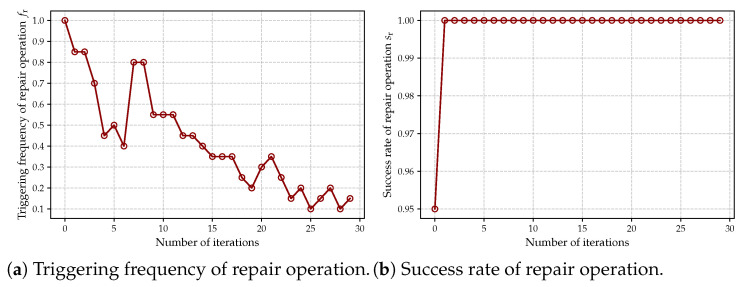
Triggering frequency and success rate of repair operation during optimization process.

First, we evaluated the trend of the triggering frequency and success rate of the repair mechanism with the number of iterations during the optimization process, as shown in Figure 11. As shown in Figure 11a, the initial solutions generated by the random initialization strategy are unable to satisfy the problem constraints, leading to a high frequency of repair operation activation in the early optimization phase. As the iterative process progresses, the clustering results gradually converge toward optimal configurations, accompanied by a steady decline in the frequency of constraint violations requiring repair. Notably, sporadic spikes in the repair frequency curve are observed during later iterations. These transient increases can be attributed to the stochastic nature of the simulated annealing mechanism, which may occasionally perturb existing solutions to generate new candidate solutions that violate constraints, thereby necessitating additional repair operations. As shown in Figure 11b, the repair mechanism consistently maintains a high success rate throughout the iterative optimization process, demonstrating its capability to effectively identify infeasible solutions and restore their constraint satisfaction. This characteristic verifies the enhancement of the repair mechanism on the robustness of the algorithm’s search procedure.

**Figure 12:**
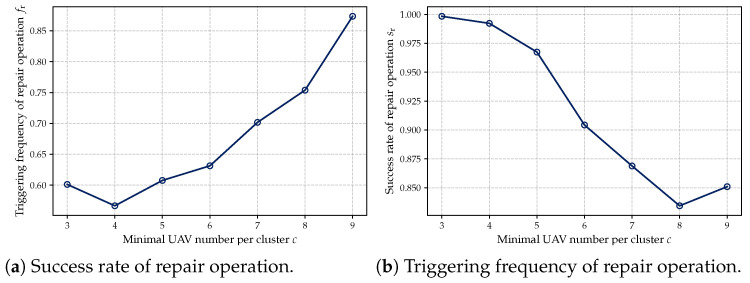
Triggering frequency and success rate of repair operation for different *c*.

**Figure 13:**
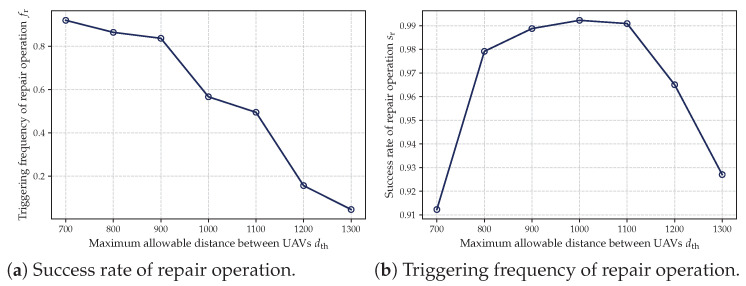
Triggering frequency and success rate of repair operation for different $d_{\mathrm{th}}$.

Second, we conducted a quantitative analysis of the repair mechanism’s behavior under different constraint levels. Specifically, two sets of experiments were designed: (1) varying the minimum number of UAVs per cluster *c* from 3 to 9, and (2) varying the maximum allowable distance between UAVs in a cluster $d_{\mathrm{th}}$ from 700 to 1300. For each configuration, we recorded the trigger frequency and success rate of the repair process. The corresponding results are illustrated in Figure 12 and Figure 13. As shown in Figure 12a and Figure 13a, larger values of *c* and smaller values of $d_{\mathrm{th}}$ represent more stringent constraints, under which the repair mechanism is triggered more frequently. Meanwhile, Figure 12b and Figure 13b demonstrate that the repair mechanism consistently maintains a high success rate across different constraint levels, indicating its robustness and effectiveness.

#### 5.3.6. Computational Complexity Analysis

We provide a systematic analysis of the computational complexity of MDQPSO-ASA in terms of the number of UAVs *N*, NCTs *M*, swarms *S* and particle swarm size *P*.

First, the DQPSO module dominates when updating particle positions. At each iteration, computing the mean position, updating each particle and performing discretization requires O(P·N) operations per swarm, leading to an overall cost of O(S·P·N) per iteration.

Second, the ASA module introduces additional overhead when refining solutions. Every $\tau_{1}$ iterations, ASA generates perturbed solutions and evaluates their energy differences in O(P·N·M) total operations per swarm, which amortizes to $O(S\cdot P\cdot N\cdot M/\tau_{1})$ per iteration.

Third, the repair mechanism, which enforces intra-cluster distance constraints via a global greedy strategy, becomes the key bottleneck. Assuming UAVs are uniformly distributed across *M* clusters, computing and sorting all pairwise distances within each cluster incurs $O({\left(N/M\right)}^{2}\log\left(N/M\right))$ per cluster; summing over *M* clusters and *S* swarms yields approximately $O\left(S\cdot N^{2}/M\cdot \log\left(N/M\right)\right)$ per iteration. Then, the repair stage asymptotically determines the overall per-iteration complexity of MDQPSO-ASA as

(38) $$O\left(S\cdot \left(\frac{N^{2}}{M}\log\frac{N}{M}+N\cdot M\right)\right).$$

Fourth, the migration operation, which is invoked every $\tau_{2}$ iterations to exchange particles among swarms, adds an amortized cost of $O(S\cdot P/\tau_{2})$ per iteration.

By integrating all these parts, the overall time complexity of the MDQPSO-ASA algorithm is

(39) $$O\left(S\cdot P\cdot N+\frac{S\cdot P\cdot N\cdot M}{\tau_{1}}+S\cdot \left(\frac{N^{2}}{M}\log\frac{N}{M}+N\cdot M\right)+\frac{S\cdot P}{\tau_{2}}\right).$$

Given that in typical cooperative localization scenarios the number of UAVs *N* is significantly larger than the number of NCTs *M*, i.e., N≫M, the first stage of the repair mechanism largely determines the overall computational complexity per iteration in practical applications. Therefore, the asymptotic time complexity of the algorithm is given by

(40) $$O\left(\frac{S\cdot N^{2}}{M}\log\frac{N}{M}\right).$$

#### 5.3.7. Runtime Analysis for Different Numbers of UAVs and NCTs

To evaluate the efficiency of the proposed algorithm, we compare the runtime of different algorithms under varying scales of UAVs and NCTs. Table 2 presents the runtime results for scenarios with a fixed number of NCTs (M=8) and different numbers of UAVs.

The results show that while our algorithm (MDQPSO-ASA) is more complex, it achieves a balance between solution quality and runtime. Compared to other PSO-based algorithms, MDQPSO-ASA does not have a significant disadvantage in terms of runtime but consistently finds better solutions, as described in Section 5.3.3. On the other hand, it shows a substantial advantage over SA and SA-GA in both runtime and solution quality. Additionally, we also compared the runtime of the algorithms with a fixed number of UAVs and different numbers of NCTs. The experiments indicate that the runtime does not significantly change with different numbers of NCTs, demonstrating the robustness of our algorithm in different scenarios.

### 5.4. Discussion on Real-World Applications

Real-world UAV systems face significant challenges, including computational constraints, limited communication bandwidth, and environmental uncertainties. Regarding these issues, the proposed MDQPSO-ASA algorithm offers several key features. First, it ensures computational efficiency through a multi-swarm framework that supports parallelization, leverages hardware acceleration (e.g., via GPUs), and allows early termination with feasible solutions via a repair mechanism. Second, it optimizes communication by employing localized coordination to minimize global communication overhead, focusing on critical metrics (e.g., CRLB and velocity vectors). Third, the algorithm demonstrates robustness to noise by incorporating CRLB derivation that accounts for measurement noise and motion-induced errors. Notably, the MDQPSO-ASA algorithm allows extensions toward heterogeneous UAV clusters by considering diverse attributes (e.g., different error covariances of sensors and time thresholds for link stability) across UAV individuals. By incorporating these heterogeneous attributes into the cluster formation and optimization processes, the algorithm can better exploit the strengths of different UAV individuals, thereby enhancing overall system performance and adaptability in complex environments.

In addition, the MDQPSO-ASA framework can be adapted to semi-centralized scenarios where UAVs are intermittently connected to ground control stations. In such settings, the ground control stations could periodically collect UAV status information and use these data to perform centralized optimization using MDQPSO-ASA. The optimized clustering decisions, including task assignments and communication topologies, could then be redistributed back to the UAVs for execution. This approach not only leverages the computational power of ground stations to solve complex optimization problems but also reduces the onboard computational burden on UAVs, thereby conserving their limited energy resources. Furthermore, by allowing intermittent connectivity, this semi-centralized adaptation effectively balances global coordination and local autonomy, making the system more resilient to dynamic environmental changes, communication disruptions, and scalability challenges. Such a hybrid architecture could be particularly advantageous in large-scale UAV networks, where fully centralized control may become impractical due to communication overhead, while fully decentralized approaches may struggle to achieve optimal performance.

## 6. Conclusions

This paper establishes a novel dynamic clustering-based optimization model for multi-to-multi target localization in UAV clusters, followed by the development of the MDQPSO-ASA algorithm to solve this complex problem. The proposed MDQPSO-ASA algorithm demonstrates robust performance through its ability to balance exploration and exploitation while adhering to binary constraints. Experiment results demonstrate the effectiveness of the proposed algorithm in enhancing localization accuracy and system performance. Future research could extend this model to incorporate heterogeneous UAVs or explore scalability in larger-scale networks.

## Figures and Tables

**Table 1:** Default parameter settings for the simulation experiments.

Parameters	Values	Descriptions
*c*	4	Minimum number of UAVs per cluster, since TOA localization model in 3D space requires at least 4 sensor nodes [27].
$d_{\mathrm{th}}$	1000	Maximum allowable distance between UAVs in a cluster.
$\alpha_{1}$	1.0	Weight factor for CRLB in the objective function.
$\alpha_{2}$	0.5	Weight factor for motion similarity in the objective function.
$\alpha_{3}$	0.5	Weight factor for communication link consistency in the objective function.
β	0.5	Acceleration coefficient in DQPSO.
$T_{0}$	100	Initial temperature for simulated annealing.
$r_{0}$	0.01	Initial cooling rate for simulated annealing.
$\tau_{1}$	5	Interval for applying simulated annealing (iterations).
$\tau_{2}$	5	Interval for migration operation (iterations).

**Table 2:** Runtime (s) of different algorithms for different numbers of UAVs with M=8 NCTs.

Number of UAVs	SA	SA-GA	DPSO	QPSO	MDQPSO	MDQPSO-ASA
40	4.866	14.663	3.100	3.159	2.970	3.304
50	13.638	31.029	9.121	8.876	8.563	9.362
60	18.946	53.495	12.682	12.935	12.144	12.818
70	21.234	82.977	16.713	16.505	15.187	16.895
80	25.696	95.775	19.270	19.211	18.917	19.715
90	27.772	122.977	22.431	22.546	21.461	23.612
100	32.818	150.155	30.220	30.519	29.588	32.520
110	34.325	165.012	34.999	34.830	32.036	35.032

## Data Availability

Data are contained within the article.

## Abbreviations

The following abbreviations are used in this manuscript:

UAV	Unmanned Aerial Vehicle
NCT	Non-Cooperative Target
CRLB	Cramér–Rao Lower Bound
CPDF	Conditional Probability Density Function
FIM	Fisher Information Matrix
TOA	Time of Arrival
SA	Simulated Annealing
ASA	Adaptive Simulated Annealing
PSO	Particle Swarm Optimization
DPSO	Discrete Particle Swarm Optimization
QPSO	Quantum-Inspired Particle Swarm Optimization
MDQPSO	Multi-Swarm Discrete Quantum-Inspired Particle Swarm Optimization
**Symbols**	**Definitions**
U, G	Set of UAVs/NCTs.
$p_{n}$, $q_{m}$	Position of UAV $u_{n}$, NCT $g_{m}$.
$v_{n}$	Velocity of UAV $u_{n}$.
$C_{k}$, $I_{k}$	*k*-th cluster, index set of UAVs in $C_{k}$.
$I_{k}^{\left(2\right)}$	Set of 2-element unordered pair in $I_{k}$.
$x_{n,k}$	Indicator if UAV $u_{n}$ is in cluster $C_{k}$.
$y_{k,m}$	Indicator if cluster $C_{k}$ is assigned to NCT $g_{m}$.
$d_{i,j}$	Distance between UAV $u_{i}$ and UAV $u_{j}$.
$D_{i,j}$, $V_{i,j}$	Position/velocity similarity between UAV $u_{i}$ and UAV $u_{j}$.
$d_{k}$	Diameter of cluster $C_{k}$.
$D_{k}$, $V_{k}$, $\Phi_{k}$	Position/velocity/motion similarity of cluster $C_{k}$.
$W_{i,j}$	Communication link consistency between UAV $u_{i}$ and UAV $u_{j}$.
$W_{k}$	Communication link consistency of cluster $C_{k}$.
$r_{m,n}$	Ideal distance between UAV $u_{n}$ and NCT $g_{m}$.
${\hat{r}}_{m,n}$	Noisy distance estimate between UAV $u_{n}$ and NCT $g_{m}$.
$\delta_{m,n}$	Total observed localization error for NCT *m* by UAV *n*.
$\Sigma_{m,n}$	Total covariance matrix of observed position error.
J	FIM.
$B_{k,m}$	CRLB for NCT *m* by cluster $C_{k}$.
